# Crystal structure of 2-chloro-1-(6-fluoro-3,4-di­hydro-2*H*-chromen-2-yl)ethanone

**DOI:** 10.1107/S1600536814019746

**Published:** 2014-09-06

**Authors:** Zheng Shen, Qiu-Xia Mao, Ji-Long Ge, Yong-Rui Tu, Yan Wang

**Affiliations:** a Changzhou Siyao Pharmacy Limited Company, Changzhou 213004, People’s Republic of China

**Keywords:** crystal structure, chromene, di­hydro­pyran ring, hydrogen bonding, dimer formation, nebivolol inter­mediate

## Abstract

In the title mol­ecule, C_11_H_10_ClFO_2_, the benzene ring, the F atom and the O atom of the di­hydro­pyran ring are essentially coplanar, with an r.m.s. deviation of 0.007 Å. The di­hydro­pyran ring is in a half-chair conformation. In the crystal, mol­ecules are linked by pairs of weak C—H⋯π hydrogen bonds, forming inversion dimers.

## Related literature   

For the application of the title compound as a key inter­mediate in the preparation of nebivolol, which is useful in treating essential hypertension, see: Raffaella *et al.* (2011 ▶).
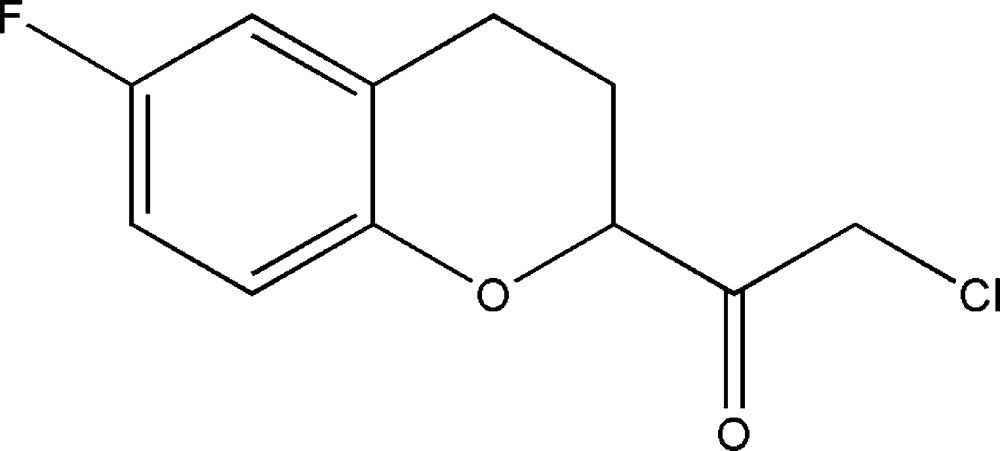


## Experimental   

### Crystal data  

C_11_H_10_ClFO_2_
*M* *_r_* = 228.64
Monoclinic, 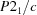

*a* = 9.704 (3) Å
*b* = 9.720 (3) Å
*c* = 10.804 (4) Å
β = 101.637 (7)°
*V* = 998.2 (6) Å^3^
*Z* = 4
Mo *K*α radiation
μ = 0.37 mm^−1^
*T* = 296 K
0.20 × 0.20 × 0.20 mm

### Data collection  

Rigaku SCXmini diffractometer
Absorption correction: multi-scan (*CrystalClear*; Rigaku, 2005 ▶) *T*_min_ = 0.983, *T*_max_ = 0.983
5810 measured reflections
1940 independent reflections
1701 reflections with *I* > 2σ(*I*)
*R*_int_ = 0.037

### Refinement  

*R*[*F* ^2^ > 2σ(*F* ^2^)] = 0.058
*wR*(*F* ^2^) = 0.169
*S* = 1.06
1940 reflections
136 parameters
H-atom parameters constrained
Δρ_max_ = 0.87 e Å^−3^
Δρ_min_ = −0.61 e Å^−3^

### 

Data collection: *CrystalClear* (Rigaku, 2005 ▶); cell refinement: *CrystalClear*; data reduction: *CrystalClear*; program(s) used to solve structure: *SHELXS97* (Sheldrick, 2008 ▶); program(s) used to refine structure: *SHELXL97* (Sheldrick, 2008 ▶); molecular graphics: *DIAMOND* (Brandenburg & Putz, 2005 ▶); software used to prepare material for publication: *SHELXL97*.

## Supplementary Material

Crystal structure: contains datablock(s) I, New_Global_Publ_Block. DOI: 10.1107/S1600536814019746/lh5719sup1.cif

Structure factors: contains datablock(s) I. DOI: 10.1107/S1600536814019746/lh5719Isup2.hkl

Supporting information file. DOI: 10.1107/S1600536814019746/lh5719Isup3.cml

**Fig. 1.**
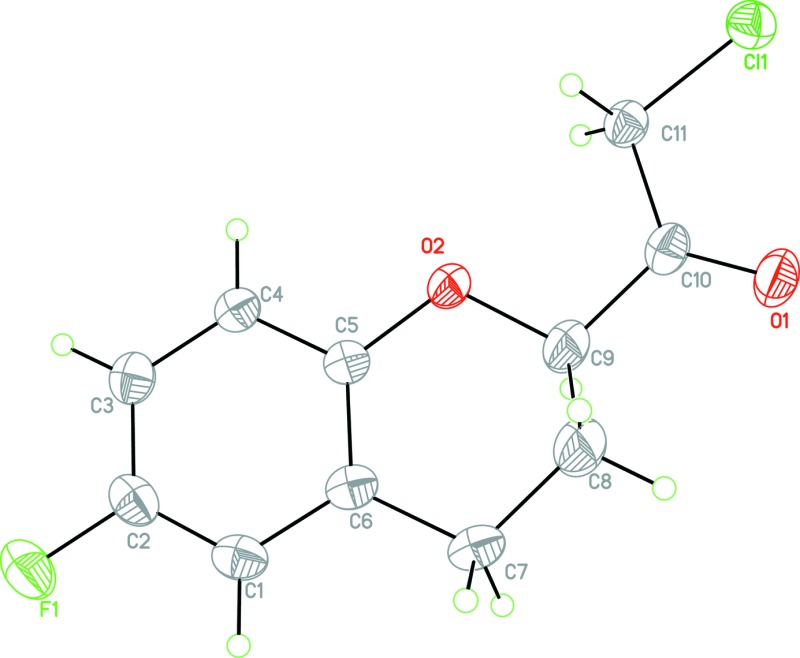
The molecular structure of the title compound with displacement ellipsoids drawn at the 30% probability level. H atoms are represented as small spheres of arbitrary radii.

**Fig. 2.**
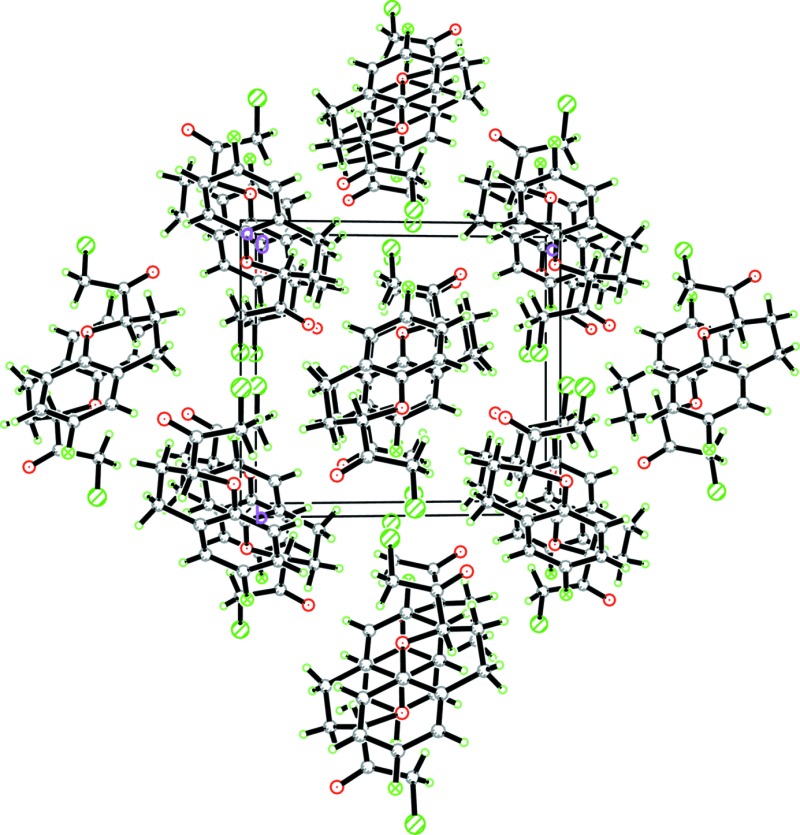
Part of the crystal structure viewed along the a axis.

. DOI: 10.1107/S1600536814019746/lh5719fig1.tif
The mol­ecular structure of the title compound with displacement ellipsoids drawn at the 30% probability level. H atoms are represented as small spheres of arbitrary radii.

a . DOI: 10.1107/S1600536814019746/lh5719fig2.tif
Part of the crystal structure viewed along the *a* axis.

CCDC reference: 992910

Additional supporting information:  crystallographic information; 3D view; checkCIF report

## Figures and Tables

**Table 1:** Hydrogen-bond geometry (Å, °) *Cg* is the centroid of the C1–C6 ring.

*D*—H⋯*A*	*D*—H	H⋯*A*	*D*⋯*A*	*D*—H⋯*A*
C11—H11*B*⋯*Cg* ^i^	0.97	2.76	3.457 (3)	129

Symmetry code: (i) 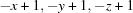.

## supplementary crystallographic information

### S1. Comment

The title compound is a key intermediate in preparating nebivolol, which is useful in treating essential hypertension (Raffaella, *et al.*, 2011). As part of our interest in these types of materials, we report herein the crystal structure of the title compound.

The molecular structure of the title compound is shown in Fig.1. Atoms F1 and O2 atoms are approximately coplanar with the benzene ring, with an r.m.s deviation of 0.007Å. The dihydropyran ring is in a half-chair conformation. In the crystal, molecules are linked by pairs of weak C—H···π hydrogen bonds forming inversion dimers (Fig. 2).

### S2. Experimental

The title compound was provided by Changzhou Siyao Pham, Ltd (Changzhou, Jiangsu). Crystals of it suitable for X-ray diffraction were obstained by slow evaporation of a methanol solution.

### S3. Refinement

All H atoms were positioned geometrically and treated as riding with C—H = 0.93 Å (aryl), C—H = 0.97 Å (methylene) and C—H = 0.98 Å (methine) with *U*_iso_(H) = 1.2*U*_eq_.

### Crystal data

**Table:**

C_11_H_10_ClFO_2_	*F*(000) = 472
*M**_r_* = 228.64	*D*_x_ = 1.521 Mg m^−^^3^
Monoclinic, *P*2_1_/*c*	Mo *K*α radiation, λ = 0.71073 Å
Hall symbol: -P 2ybc	Cell parameters from 1940 reflections
*a* = 9.704 (3) Å	θ = 2.1–26.0°
*b* = 9.720 (3) Å	µ = 0.37 mm^−^^1^
*c* = 10.804 (4) Å	*T* = 296 K
β = 101.637 (7)°	Prism, colourless
*V* = 998.2 (6) Å^3^	0.20 × 0.20 × 0.20 mm
*Z* = 4	

### Data collection

**Table:**

Rigaku SCXmini diffractometer	1940 independent reflections
Radiation source: fine-focus sealed tube	1701 reflections with *I* > 2σ(*I*)
Graphite monochromator	*R*_int_ = 0.037
Detector resolution: 13.6612 pixels mm^-1^	θ_max_ = 26.0°, θ_min_ = 2.1°
CCD_Profile_fitting scans	*h* = −11→11
Absorption correction: multi-scan (*CrystalClear*; Rigaku, 2005)	*k* = −11→11
*T*_min_ = 0.983, *T*_max_ = 0.983	*l* = −12→13
5810 measured reflections	

### Refinement

**Table:**

Refinement on *F*^2^	Primary atom site location: structure-invariant direct methods
Least-squares matrix: full	Secondary atom site location: difference Fourier map
*R*[*F*^2^ > 2σ(*F*^2^)] = 0.058	Hydrogen site location: inferred from neighbouring sites
*wR*(*F*^2^) = 0.169	H-atom parameters constrained
*S* = 1.06	*w* = 1/[σ^2^(*F*_o_^2^) + (0.087*P*)^2^ + 0.9962*P*] where *P* = (*F*_o_^2^ + 2*F*_c_^2^)/3
1940 reflections	(Δ/σ)_max_ < 0.001
136 parameters	Δρ_max_ = 0.87 e Å^−^^3^
0 restraints	Δρ_min_ = −0.61 e Å^−^^3^

### Special details

**Table:**

Geometry. All e.s.d.'s (except the e.s.d. in the dihedral angle between two l.s. planes) are estimated using the full covariance matrix. The cell e.s.d.'s are taken into account individually in the estimation of e.s.d.'s in distances, angles and torsion angles; correlations between e.s.d.'s in cell parameters are only used when they are defined by crystal symmetry. An approximate (isotropic) treatment of cell e.s.d.'s is used for estimating e.s.d.'s involving l.s. planes.
Refinement. Refinement of *F*^2^ against ALL reflections. The weighted *R*-factor *wR* and goodness of fit *S* are based on *F*^2^, conventional *R*-factors *R* are based on *F*, with *F* set to zero for negative *F*^2^. The threshold expression of *F*^2^ > σ(*F*^2^) is used only for calculating *R*-factors(gt) *etc*. and is not relevant to the choice of reflections for refinement. *R*-factors based on *F*^2^ are statistically about twice as large as those based on *F*, and *R*- factors based on ALL data will be even larger.

### Fractional atomic coordinates and isotropic or equivalent isotropic displacement parameters (Å^2^)

**Table:**

	*x*	*y*	*z*	*U*_iso_*/*U*_eq_	
Cl1	0.29999 (8)	0.06003 (8)	0.46282 (7)	0.0520 (3)	
O2	0.6337 (2)	0.3728 (2)	0.50089 (17)	0.0446 (5)	
F1	1.0157 (2)	0.7779 (2)	0.4805 (2)	0.0693 (6)	
C5	0.7331 (3)	0.4738 (3)	0.5024 (2)	0.0370 (6)	
C1	0.9201 (3)	0.6183 (3)	0.6021 (3)	0.0465 (7)	
H1	0.9827	0.6483	0.6738	0.056*	
C6	0.8247 (3)	0.5141 (3)	0.6117 (3)	0.0399 (6)	
C4	0.7381 (3)	0.5330 (3)	0.3864 (3)	0.0415 (6)	
H4	0.6773	0.5026	0.3137	0.050*	
C3	0.8324 (3)	0.6361 (3)	0.3789 (3)	0.0476 (7)	
H3	0.8355	0.6776	0.3019	0.057*	
C7	0.8214 (3)	0.4463 (3)	0.7360 (3)	0.0504 (7)	
H7A	0.9162	0.4203	0.7767	0.060*	
H7B	0.7865	0.5112	0.7906	0.060*	
C11	0.4282 (3)	0.1895 (3)	0.4699 (3)	0.0433 (6)	
H11A	0.4958	0.1617	0.4197	0.052*	
H11B	0.3834	0.2735	0.4335	0.052*	
C10	0.5040 (3)	0.2175 (3)	0.6024 (3)	0.0458 (7)	
C2	0.9219 (3)	0.6765 (3)	0.4875 (3)	0.0483 (7)	
O1	0.4794 (3)	0.1587 (3)	0.6926 (2)	0.0607 (6)	
C9	0.6086 (4)	0.3338 (5)	0.6206 (3)	0.0700 (11)	
H9	0.5568	0.4116	0.6465	0.084*	
C8	0.7298 (5)	0.3218 (5)	0.7187 (4)	0.0829 (14)	
H8A	0.7849	0.2438	0.7006	0.099*	
H8B	0.6999	0.3028	0.7974	0.099*	

### Atomic displacement parameters (Å^2^)

**Table:**

	*U*^11^	*U*^22^	*U*^33^	*U*^12^	*U*^13^	*U*^23^
Cl1	0.0554 (5)	0.0483 (5)	0.0536 (5)	−0.0019 (3)	0.0140 (3)	−0.0035 (3)
O2	0.0478 (11)	0.0536 (12)	0.0315 (9)	−0.0070 (9)	0.0054 (8)	0.0040 (8)
F1	0.0627 (12)	0.0574 (12)	0.0896 (15)	−0.0171 (10)	0.0197 (11)	−0.0058 (11)
C5	0.0363 (13)	0.0358 (13)	0.0388 (14)	0.0062 (10)	0.0070 (11)	−0.0012 (10)
C1	0.0396 (14)	0.0454 (16)	0.0528 (16)	0.0045 (12)	0.0052 (12)	−0.0122 (13)
C6	0.0376 (13)	0.0417 (14)	0.0398 (14)	0.0112 (11)	0.0062 (11)	−0.0056 (11)
C4	0.0409 (14)	0.0452 (15)	0.0380 (14)	0.0029 (11)	0.0073 (11)	−0.0007 (11)
C3	0.0486 (16)	0.0464 (16)	0.0505 (16)	0.0062 (13)	0.0159 (13)	0.0041 (13)
C7	0.0517 (17)	0.0602 (19)	0.0357 (14)	0.0038 (14)	0.0007 (12)	−0.0048 (13)
C11	0.0477 (15)	0.0429 (14)	0.0399 (14)	0.0034 (12)	0.0100 (12)	0.0038 (11)
C10	0.0455 (15)	0.0534 (17)	0.0388 (14)	0.0048 (13)	0.0091 (12)	0.0093 (12)
C2	0.0417 (15)	0.0381 (14)	0.067 (2)	−0.0003 (11)	0.0159 (14)	−0.0067 (13)
O1	0.0602 (13)	0.0790 (16)	0.0427 (12)	−0.0096 (12)	0.0101 (10)	0.0160 (11)
C9	0.078 (2)	0.092 (3)	0.0366 (16)	−0.028 (2)	0.0032 (15)	0.0125 (17)
C8	0.088 (3)	0.108 (3)	0.0448 (19)	−0.035 (3)	−0.0046 (18)	0.022 (2)

### Geometric parameters (Å, º)

**Table:**

Cl1—C11	1.761 (3)	C3—H3	0.9300
O2—C5	1.374 (3)	C7—C8	1.492 (5)
O2—C9	1.416 (4)	C7—H7A	0.9700
F1—C2	1.354 (3)	C7—H7B	0.9700
C5—C6	1.383 (4)	C11—C10	1.497 (4)
C5—C4	1.389 (4)	C11—H11A	0.9700
C1—C2	1.364 (5)	C11—H11B	0.9700
C1—C6	1.391 (4)	C10—O1	1.194 (4)
C1—H1	0.9300	C10—C9	1.506 (5)
C6—C7	1.501 (4)	C9—C8	1.420 (5)
C4—C3	1.370 (4)	C9—H9	0.9800
C4—H4	0.9300	C8—H8A	0.9700
C3—C2	1.370 (5)	C8—H8B	0.9700
			
C5—O2—C9	115.5 (2)	C10—C11—H11A	109.2
O2—C5—C6	122.7 (2)	Cl1—C11—H11A	109.2
O2—C5—C4	115.9 (2)	C10—C11—H11B	109.2
C6—C5—C4	121.3 (3)	Cl1—C11—H11B	109.2
C2—C1—C6	120.1 (3)	H11A—C11—H11B	107.9
C2—C1—H1	120.0	O1—C10—C11	123.6 (3)
C6—C1—H1	120.0	O1—C10—C9	119.5 (3)
C5—C6—C1	117.7 (3)	C11—C10—C9	116.7 (2)
C5—C6—C7	120.9 (3)	F1—C2—C1	119.0 (3)
C1—C6—C7	121.4 (3)	F1—C2—C3	118.6 (3)
C3—C4—C5	120.1 (3)	C1—C2—C3	122.4 (3)
C3—C4—H4	119.9	O2—C9—C8	115.8 (3)
C5—C4—H4	119.9	O2—C9—C10	108.5 (3)
C2—C3—C4	118.3 (3)	C8—C9—C10	118.1 (3)
C2—C3—H3	120.8	O2—C9—H9	104.2
C4—C3—H3	120.8	C8—C9—H9	104.2
C8—C7—C6	111.3 (2)	C10—C9—H9	104.2
C8—C7—H7A	109.4	C9—C8—C7	114.2 (3)
C6—C7—H7A	109.4	C9—C8—H8A	108.7
C8—C7—H7B	109.4	C7—C8—H8A	108.7
C6—C7—H7B	109.4	C9—C8—H8B	108.7
H7A—C7—H7B	108.0	C7—C8—H8B	108.7
C10—C11—Cl1	112.2 (2)	H8A—C8—H8B	107.6

### Hydrogen-bond geometry (Å, º)

Cg is the centroid of the C1–C6 ring.

**Table:**

*D*—H···*A*	*D*—H	H···*A*	*D*···*A*	*D*—H···*A*
C11—H11*B*···*Cg*^i^	0.97	2.76	3.457 (3)	129

Symmetry code: (i) −*x*+1, −*y*+1, −*z*+1.
