# Supplementary figures and images for: Crystal structure of 2-chloro-1-(6-fluoro-3,4-di­hydro-2H-chromen-2-yl)ethanone
Source: Acta Crystallogr Sect E Struct Rep Online. 2014 Sep 6;70(Pt 10):o1087. doi: 10.1107/S1600536814019746 (PMC4257220; doi:10.1107/S1600536814019746)

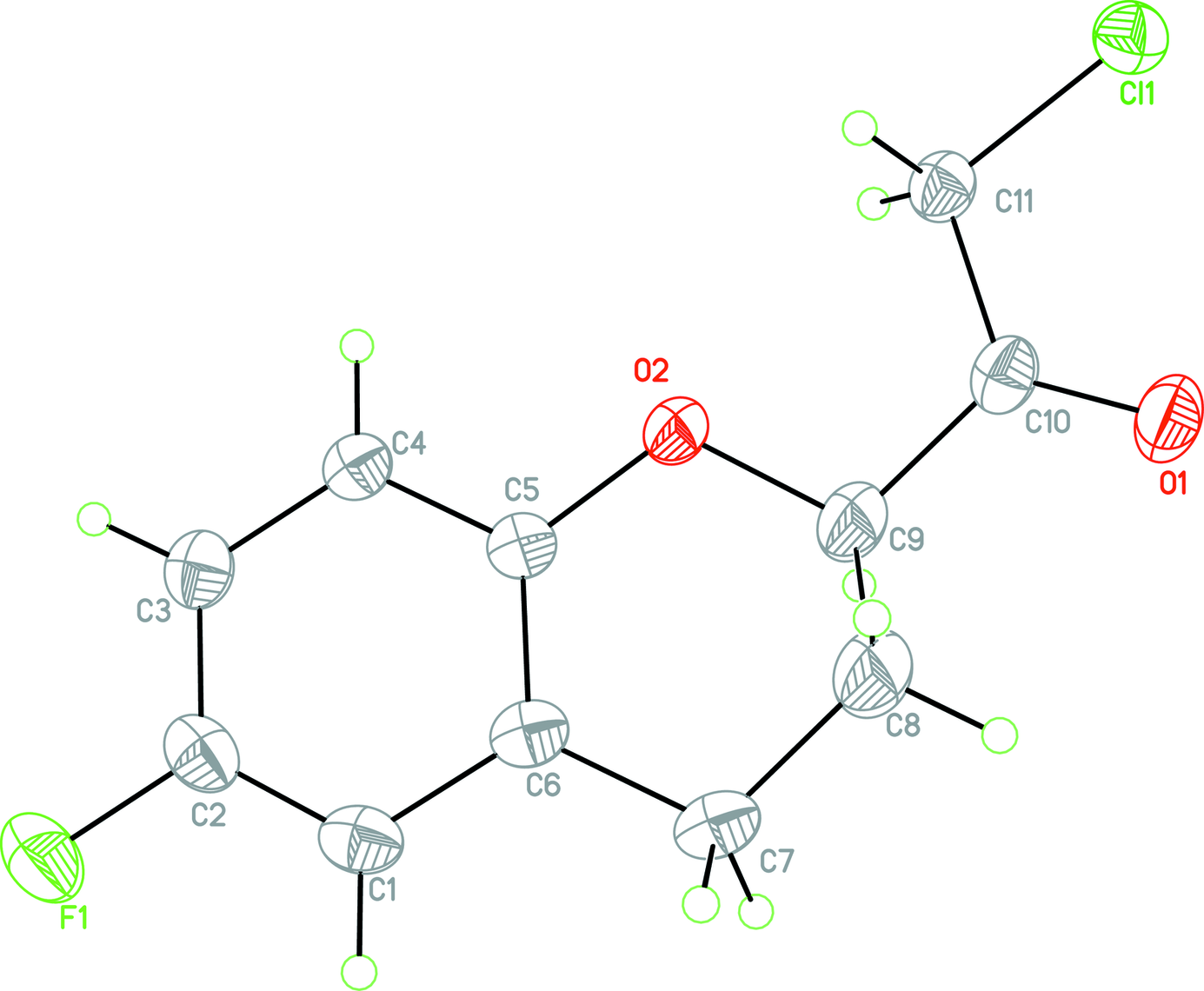

Supplement: Supplementary file 4 [file e-70-o1087-fig1.tif]

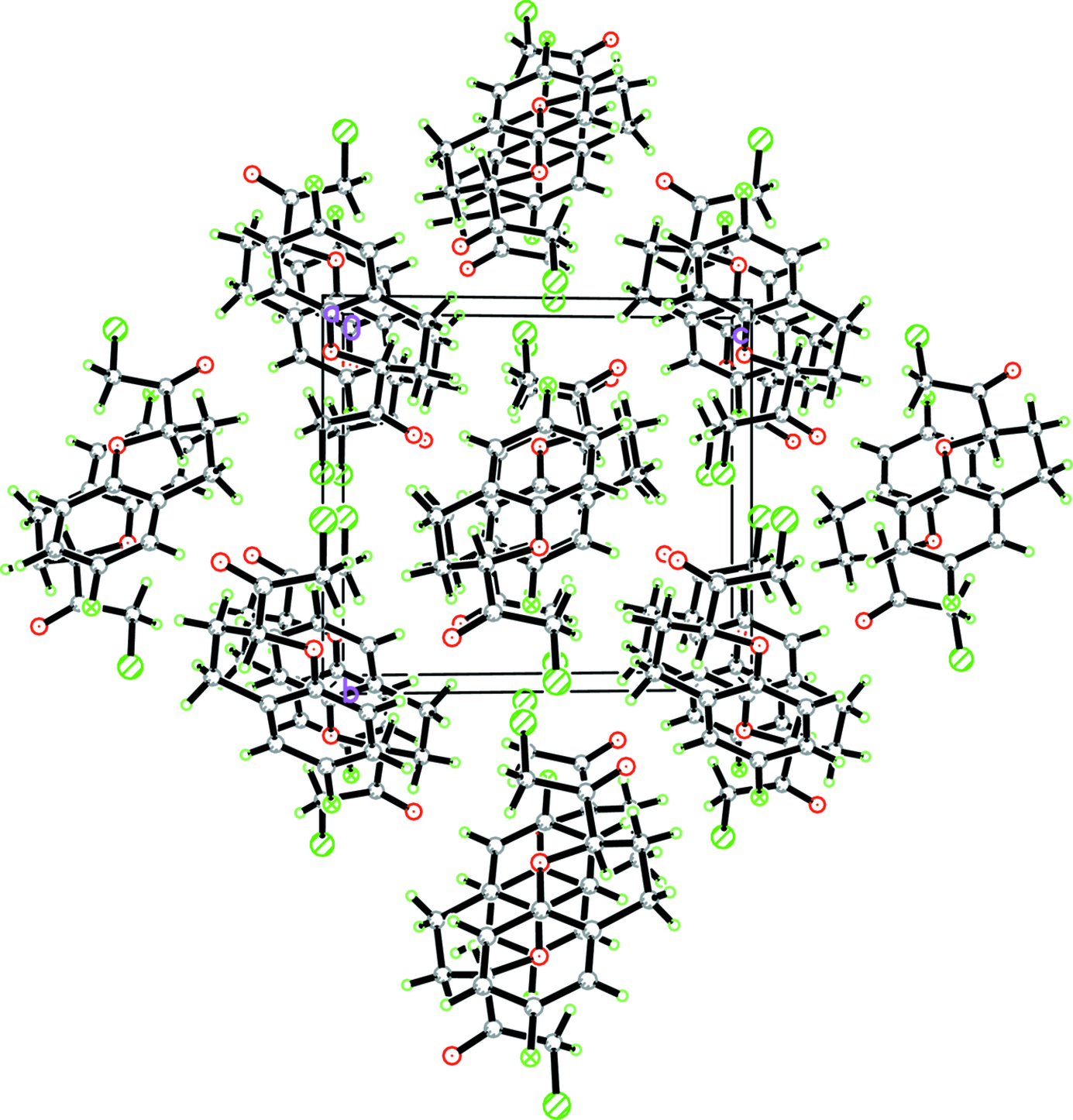

Supplement: Supplementary file 5 [file e-70-o1087-fig2.tif]
